# Thrombotic thrombocytopenic purpura and systemic lupus erythematosus: Successful management of a rare presentation

**DOI:** 10.4103/0972-5229.43682

**Published:** 2008

**Authors:** Pratish George, Jasmine Das, Basant Pawar, Naveen Kakkar

**Affiliations:** **From:** Department of Internal Medicine, Christian Medical College and Hospital, Ludhiana, Punjab, India; 1 Department of Nephrology, Christian Medical College and Hospital, Ludhiana, Punjab, India; 2 Department of Pathology, Christian Medical College and Hospital, Ludhiana, Punjab, India

**Keywords:** Plasma exchange, systemic lupus erythematosus, thrombotic thrombocytopenic purpura

## Abstract

Thrombotic thrombocytopenic purpura (TTP) and systemic lupus erythematosus (SLE) very rarely present simultaneously and pose a diagnostic and therapeutic dilemma to the critical care team. Prompt diagnosis and management with plasma exchange and immunosuppression is life-saving. A patient critically ill with TTP and SLE, successfully managed in the acute period of illness with plasma exchange, steroids and mycophenolate mofetil is described.

## Introduction

Systemic lupus erythematosis (SLE) is diagnosed by the presence of four or more of the following criteria, serially or simultaneously: malar rash, discoid rash, photosensitivity, oral ulcers, non erosive arthritis, serositis, renal abnormalities including proteinuria or active urinary sediments, neuropsychiatric features, hematological abnormalities including hemolytic anemia, leucopenia, lymphopenia and thrombocytopenia, immunological markers like anti-ds DNA or anti-Smith antibody and high Antinuclear antibody titres. Thrombotic thrombocytopenic purpura (TTP) in patients with SLE is extremely rare. The overall incidence of TTP in SLE patients is unclear and has been reported to be as low as 0.5%. A review includes about 40 cases of TTP related to SLE.[1] Very rarely TTP presents simultaneously with SLE in ICU.

## Case Report

A 30-year-old lady was admitted with fever and jaundice. A week earlier she had undergone an uncomplicated medical termination of pregnancy at another hospital, at 13 weeks of gestation. She had an uneventful pregnancy with twins two years earlier and the twins were diagnosed to have thalassemia major. She was subsequently diagnosed to have thalassemia minor and her husband had thalassemia minor trait. No earlier history of spontaneous first trimester abortions was present.

She had weight loss and alopecia for a year with a rash noticed on her face and limbs for 12 weeks. No neurological, cardiopulmonary, gastrointestinal or urinary symptoms were present. On examination, she had non scarring alopecia and a macular, erythematous rash on her face with mild pallor and icterus. She was normotensive and had low grade pyrexia. Mild hepatosplenomegaly was detected. Respiratory,cardiovascular and neurological, evaluation was normal. Investigations prior to admission revealed normal hematological and renal parameters with mildly deranged LFT -with direct hyperbilirubinemia(Total Bilirubin- 3.3 mg/dL, Direct Bilirubin- 2.7 mg/ dL), elevated transaminases Aspartate aminotransferase- 593 U/L, Alanine aminotransferase- 73 U/L) and elevated Alkaline Phosphatase -668 U/L.

**Figure 1:**
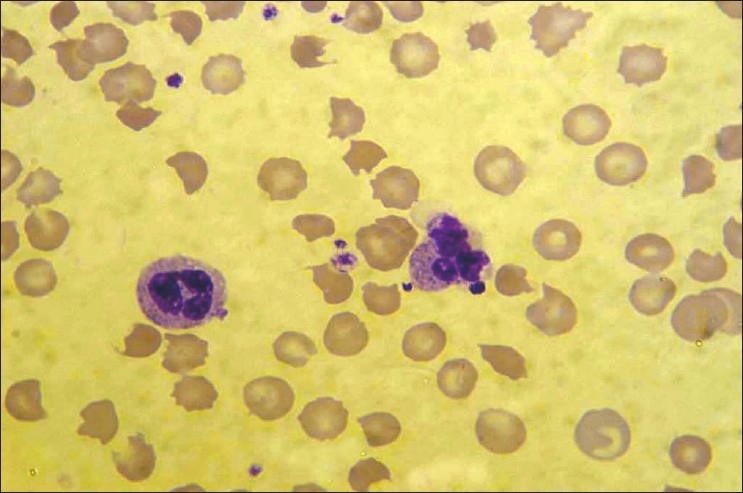
Peripheral blood film showing many schistocytes, (Leishman, ×1000)

Her condition deteriorated on the second day after admission, necessitating ICU admission with worsening jaundice and altered sensorium along with severe abdominal pain. Investigations revealed anemia, with a hemoglobin of 5.7 g/dl, reticulocytosis (corrected 3.4%), normal total and differential counts, schistocytes in the peripheral blood smear and normal prothrombin/ partial thromboplastin time [Figure 1]. Platelet count was 148000/cu.mm and lactate dehydrogenase (LDH) level was 1047 IU/dl. The serum creatinine rose to 2.3 mg/dl. Liver function showed worsening direct bilirubinemia with mild elevation of transaminases (Total Bilirubin- 13.5 mg/dL, Direct Bilirubin- 9.4 mg/ dL, Aspartate aminotransferase- 323 U/L, Alanine aminotransferase- 46 U/L.) Viral hepatitis serology for hepatitis A, B, C and E were negative Leptospira antibody was not detected. Urinanalysis showed increasing proteinuria to 3+ and 50 - 60 RBCs per high power field. Direct antiglobulin (Coomb's) test was positive. ANA and anti-DsDNA titres were high at 17.4 and 46.2 units respectively with normal complement levels. Renal and liver biopsy were not attempted in view of the patient's poor condition for the procedure.

The presence of microangiopathic anemia, alteration in neurological status, fever, thrombocytopenia and renal dysfunction with a background of malar and discoid rash, high titre of ANA and anti ds-DNA, proteinuria with active urinary sediment suggested a diagnosis of TTP and Systemic Lupus SLE. The patient was promptly treated with pulse therapy with methylprednisolone 1 gm daily for three days and plasma exchange. She underwent daily plasma exchanges with fresh frozen plasma (FFP) for five days and her sensorium improved. The platelet count rose to 229,000/cu.mm, with a reduction in the number of schistocytes in the peripheral blood smear. The LDH level also returned to the normal value. Plasma exchange was withheld. Two days later, she became stuporose, the platelet count fell to 34,000/cu.mm, renal failure worsened and she required ventilatory support. The LDH rose to 682 IU/dL. Mycophenolate mofetil was initiated thereafter in view of deteriorating clinical status with multisystem involvement and plasma exchange was continued for another seven sessions. Over the next two weeks, her sensorium improved, renal and pulmonary functions normalized, no active hemolysis was noticed and the facial rash resolved. The LDH continued to be marginally raised. She developed a ventilator associated chest infection which was treated with meropenem and satisfactory resolution over the next two weeks. The patient was discharged thereafter on prednisolone 1 mg/kg/day and mycophenolate mofetil 2 gm/day. She is in disease remission after three years, with normal renal function, on low dose prednisolone and tapering dose of mycophenolate mofetil.

## Discussion

TTP was initially described with thrombocytopenia, microangiopathic hemolytic anemia, neurologic abnormalities, renal abnormalities, and fever. There was no effective treatment and ninety percent of patients died. Plasma exchange has improved survival rates since then from 10% to between 75% and 92%, creating urgency for initiation of treatment. This has resulted in the diagnostic criteria to be revised from the earlier pentad to the current dyad of thrombocytopenia and microangiopathic hemolytic anemia, with no clinically apparent alternative explanation for thrombocytopenia and anemia.[2]

Patients with TTP have a severe deficiency of von Willebrand Factor (VWF) cleaving metalloproteinase (ADAMTS-13), which normally cleaves the unusually large(UL)VWF into smaller and less adhesive VWF,[3] resulting in microvascular thrombosis and thrombocytopenia when deficient. Connective tissue disorders like SLE have low levels of ADAMTS-13[4] suggesting a possible common pathophysiology for this disease association.

TTP occurring in patients with SLE can be difficult to diagnose because of overlapping features of the two disorders.[5] SLE may present with hemolytic anemia, thrombocytopenia, neurologic deficits, fever, and renal insufficiency but the finding of fragmented RBC's or schistocytes favours the diagnosis of TTP.[6] An initial schistocyte count of more than 1% in the absence of any other cause for thrombocytopenia is strongly suggestive of TTP.[7]

However because of the complexity in identifying schistocytes on a blood film, a wide variation is seen amongst laboratory personnel.[8] This could be disastrous for the patient if the schistocytes are not identified or diagnosis is delayed. Attempts at improving diagnosis with automation (ADVIA 120) and direct measurement of the RBC fragments have not been successful because despite the absence of false negatives, the specificity was low and had to be reconfirmed by a manual examination of the peripheral blood smear.[9] Careful examination of the blood film is therefore mandatory and specifically sensitizing the lab personnel to look for schistocytes will improve the outcomes of the disease by reducing false negatives. In our practice the peripheral blood film is immediately evaluated by a senior pathologist enabling plasma exchange to be initiated with shortest possible delay. This is important because the disease responds well to early plasma exchange treatment.

TTP in association with SLE appears to be under diagnosed. A positive Coombs test is not against the diagnosis of TTP in this setting.[5] Interestingly this patient also possibly had a rare manifestation of liver disease in SLE- cholestatic hepatitis, as evidenced by the direct hyperbilirubinemia with mild elevation of transaminases and raised alkaline phosphatase, in the absence of any infective causes for the same and gradual resolution with prednisolone.[10]

Histopathological confirmation of this suspicion was not possible.

Pregnancy often presents with a diagnostic dilemma in TTP due a similar presentation in pre eclampsia, eclampsia and HELLP (Hemolysis, Elevated Liver Enzymes, Low Platelets) syndrome. An important distinguishing feature in these conditions is an improvement post delivery.[11]

No clinical parameters predict the required duration for plasma exchange and the decision to stop plasma exchange at this time is empirical. The British Committee for Standards in Hematology (BCHS) guidelines recommends that plasma-exchange therapy be continued for a minimum of two days after the platelet count returns to normal (>150,000 /cu. mm), normal neurological status, rising hemoglobin and normal LDH. The presence of residual schistocytes on peripheral blood film after normalization of platelet counts is common and is not predictive of a relapse.[12] High dose plasma infusion may be used in an emergency till plasma exchange can be done[13] and would logically be the next best option if plasma exchange facilities are unavailable.

BCHS also recommends the use of glucocorticoids for all patients with TTP and intensive immunosuppression in severe, refractory or recurrent disease.[14] Platelet transfusion is contraindicated in TTP.[15] Cyclophosphamide has been extensively used to manage lupus nephritis but its use in fertile women is limited by premature ovarian failure and infection. Mycophenolate mofetil, an immunosuppressive agent that inhibits purine nucleotide synthesis in activated lymphocytes, is being widely accepted to be equally effective and beneficial in patients showing resistance to cyclophosphamide. It also inhibits vascular smooth muscle proliferation and atherosclerosis, which is of immense concern in patients with SLE.[16] TTP with organ dysfunction necessitating ICU admission has high mortality (35%) with good outcome in survivors. Neurological impairment is an adverse prognostic marker for mortality but not renal dysfunction.[17] Infections are not uncommon (12.8%) in patients undergoing plasma exchange[18] but infective risk due to the procedure was disapproved in a large study of patients with severe lupus nephritis on immunosupression.[19] Therefore emphasis must be given to infection prevention in the ICU with hand-washing, optimal care of indwelling catheters and preventing ventilator acquired infections, in this highly susceptible group. Once an ICU infection does take place, as in our patient, it is prudent to institute high line antibiotic support and continue immunosupression and plasma exchange, evaluating closely for deterioration with daily leucocyte counts and rising trend of acute phase reactants. No guidelines are available for modification of plasma exchange regimes in a severe infection and it should be continued due to high mortality associated with the underlying disease.

SLE presenting as TTP is rare, emphasizing the importance of looking out for the association, early diagnosis and aggressive management with plasma exchange and immunosuppression which is life-saving. Critical care specialists need to evolve an urgent multi-disciplinary approach, emphasizing on early recognition and institution of treatment to ensure better outcomes in this scenario.
